# Malignant transformation of oral lichen planus: a retrospective study of 565 Japanese patients

**DOI:** 10.1186/s12903-021-01652-7

**Published:** 2021-06-10

**Authors:** Fumihiko Tsushima, Jinkyo Sakurai, Atsushi Uesugi, Yu Oikawa, Toshimitsu Ohsako, Yumi Mochizuki, Hideaki Hirai, Kou Kayamori, Hiroyuki Harada

**Affiliations:** 1 Department of Oral and Maxillofacial Surgery, Division of Oral Health Sciences, Graduate School, Tokyo Medical and Dental University, 1-5-45 Yushima, Bunkyo-ku, Tokyo, 113-8510 Japan; 2 Department of Oral Surgery, Institute of Biomedical Sciences, Graduate School, Tokushima University, 2-50-1 Kuramoto-cho, Tokushima, 770-8503 Japan; 3 Department of Oral Pathology, Division of Oral Health Sciences, Graduate School, Tokyo Medical and Dental University, 1-5-45 Yushima, Bunkyo-ku, Tokyo, 113-8510 Japan

**Keywords:** Oral lichen planus, Oral squamous cell carcinoma, Malignant transformation, Retrospective study

## Abstract

**Background:**

Oral lichen planus (OLP) is a chronic inflammatory oral mucosa disease that is recognized as an oral potentially malignant disorder. However, the potentially malignant nature of OLP remains unclear.

**Methods:**

We designed this study to examine the demographic and clinical characteristics of patients with OLP and evaluate the associated malignant transformation rate. A total of 565 patients with a clinical and histopathological diagnosis of OLP who presented at our department between 2001 and 2017 were retrospectively studied. Patients who had clinical and histopathological features of oral lichenoid lesions (OLLs) classified as oral lichenoid contact lesions, oral lichenoid drug reactions and oral lichenoid lesions of graft-versus-host disease were excluded.

**Results:**

The study population included 123 men and 442 women aged 21–93 years (mean ± standard deviation, 60.5 ± 11.8). The 565 patients were followed up for a duration of 55.9 ± 45.3 months, during which 4 (0.7%) patients developed squamous cell carcinoma (SCC). In three of these 4 patients who developed SCC, the clinical type of OLP was the red type.

**Conclusions:**

Our results suggested that OLP was associated with a low risk of malignant transformation. We recommend regular follow-up for OLP patients and clear differentiation of oral epithelial dysplasia and OLLs to enable early detection of malignant transformation. Further investigation of the clinical risk factors associated with malignant transformation is necessary.

## Background

Oral lichen planus (OLP) is a chronic inflammatory oral mucosa disease of unknown etiology that has an estimated global prevalence of 1.01% [1]. OLP mostly occurs in middle aged persons, with a greater prevalence in females. The characteristic clinical features of OLP presents as white papules that enlarge and coalesce to form a reticular, annular or plaque-like pattern with or without atrophic or erosion [2]. There are six clinical patterns of OLP: reticular, papular, plaque, atrophic, erosive and bullous. A pigmented reticular pattern is sometimes seen [3]. The World Health Organization (WHO) Collaborating Center for Oral Cancer has defined OLP as an oral potentially malignant disorder (OPMD) [4]. However, Gonzalez-Moles et al. [5] in 2008 stated that malignant transformation of OLP is controversial mainly due to the use of varied inclusion and exclusion criteria in previous follow-up studies. In 1978, the WHO first published the clinical and histopathologic criteria for OLP diagnosis [6] that did not mention whether epithelial dysplasia was distinguished or excluded from the OLP diagnosis. In 2003, Van der Meiji and van der Waal proposed modifying the WHO diagnostic criteria [7] and confirmed the absence of epithelial dysplasia in OLP diagnosis, attempting to exclude lichenoid dysplasia from OLP. Furthermore, in 2016, the American Academy of Oral and Maxillofacial Pathology (AAOMP) proposed diagnostic criteria for OLP [3]. They emphasized clinical and histopathologic correlations in making the diagnosis of OLP. Therefore, they recommended that clinicians provide all relevant clinical information to pathologists to aid in accurate diagnosis and encouraged active discussion between clinicians and pathologists in situations of persistent doubt.

Oral lichenoid lesions (OLLs) have clinical and histopathologic similarities to OLP and have been classified as oral lichenoid contact lesions (OLCLs) caused by dental substances, oral lichenoid drug reactions (OLDRs) triggered by systemic drugs and oral lichenoid lesions of graft-versus-host disease (OLL-GVHD) at the 2006 World Workshop of Oral Medicine IV [8]. However, clear and reliable clinical and histological criteria were not obtained to fully differentiate OLLs from OLP. Recently, Carrozzo et al. [2] suggested pragmatic diagnostic criteria and a comprehensive classification of OLP and OLLs.

Six recent systematic reviews and meta-analyses have shown that the malignant transformation rate of OLP ranges from 0.44 to 1.4% [9–14]. These results showed that OLP had malignant potential; however, some of cited studies lacked the clear diagnostic criteria for OLP. These studies also listed the following as clinical risk factors for the malignant transformation of OLP: tongue localization, red type (atrophic or erosive pattern), tobacco and alcohol consumption, and hepatitis C virus (HCV) infection.

This retrospective study aimed to investigate the demographic and clinical characteristics of patients with OLP using the AAOMP proposed diagnostic criteria and evaluate the malignant potential of OLP in a Japanese cohort of patients.

## Methods

### Ethical considerations

This study was approved by the ethics committee board of the faculty of dentistry of Tokyo Medical and Dental University (D2015-575).

### Diagnostic criteria for OLP

We used the AAOMP proposed criteria for OLP in this study (Table 1). Patients were diagnosed with OLP by having their records reviewed by both experienced clinicians and experienced pathologists according to these diagnostic criteria. Patients were excluded from this study for the following reasons: (1) Any patients who were not histopathologically examined; (2) any patients who had clinical and histopathological features of OLLs proposed by the 2006 World Workshop of Oral Medicine IV [8]; and (3) any patients who were followed up for less than 6 months, even if they were diagnosed with OLP.

**Table 1:** The American Academy of Oral and Maxillofacial Pathology (AAOMP) proposed diagnostic criteria of OLP [3]

*Clinical criteria*
Multifocal symmetric distribution
White and red lesions exhibiting one or more of the following forms:
Reticular/papular
Atrophic (erythematous)
Erosive (ulcerative)
Plaque
Bullous
Lesions are not localized exclusively to the sites of smokeless tobacco placement
Lesions are not localized exclusively adjacent to and in contact with dental restorations
Lesions onset does not correlate with the start of a medication
Lesions onset does not correlate with the use of cinnamon-containing products
*Histopathological criteria*
Band-like or patchy, predominately lymphocytic infiltrate in the lamina propria confined to the epithelium-lamina propria interface
Basal cell liquefactive (hydropic) degeneration
Lymphocytic exocytosis
Absence of epithelial dysplasia
Absence of verrucous epithelial architectural change

### Patients

**Fig. 1:**
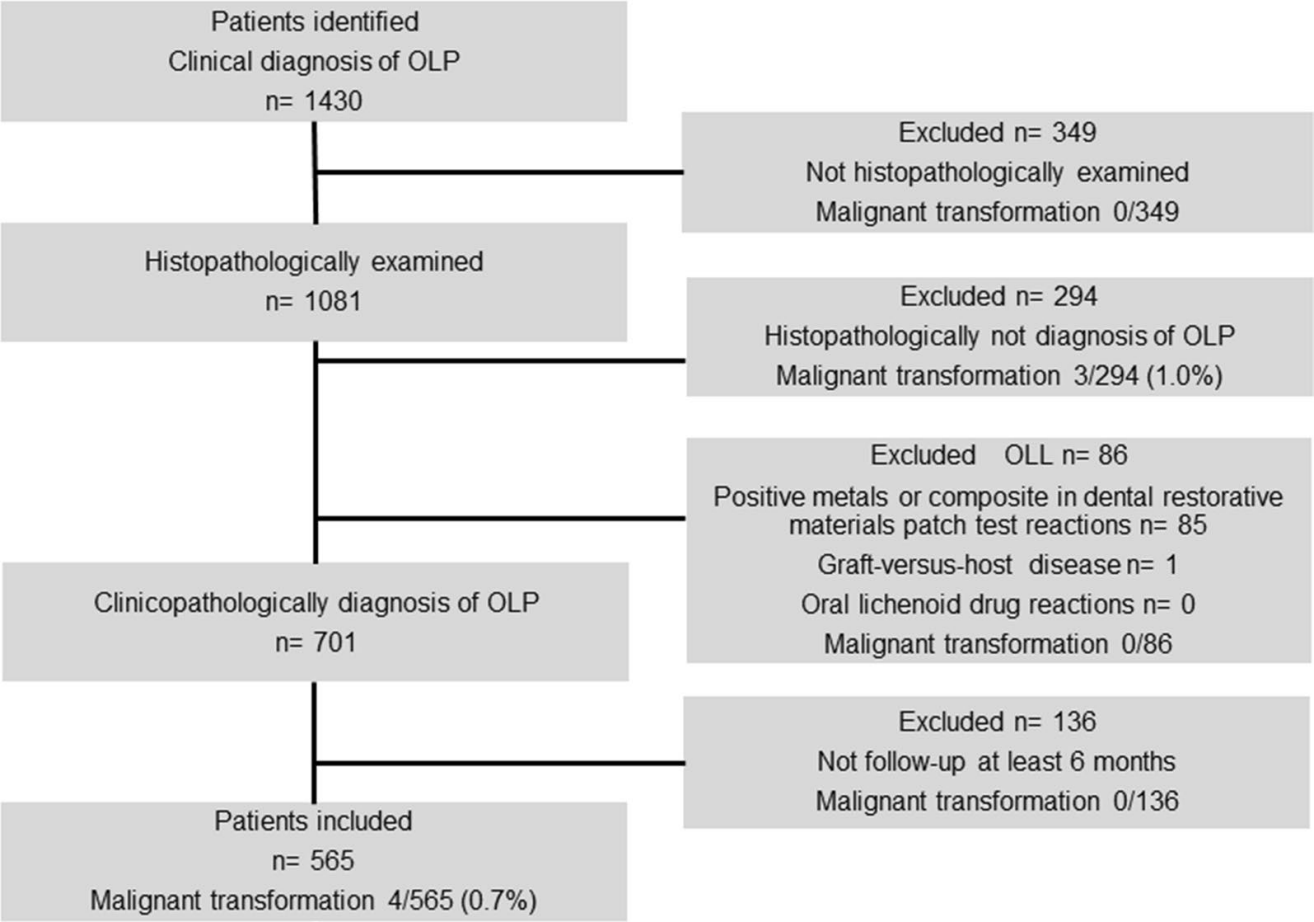
Flowchart of the patients inclusion and exclusion

We showed the flowchart of the patients inclusion and exclusion in Fig. 1. This study retrospectively analyzed the records of 1430 patients with a clinical diagnosis of OLP between 2001 and 2017. The records were accessed from the archives of the Department of Oral and Maxillofacial Surgery, Graduate School, Tokyo Medical and Dental University. Of these, 1081 patients (75.6%) were subjected to histopathological examination. Two hundred ninety-four (27.2%) patients who were not diagnosed with OLP on histopathological examination were excluded from the analyses. Details of histopathological diagnosis of not OLP patients were shown in Table 2. One (0.3%) patient was diagnosed with squamous cell carcinoma (SCC), and 81 (27.6%) were diagnosed with epithelial dysplasia or atypical epithelium; 3 (3.7%) of these 81 patients developed SCC during the follow-up. Furthermore, we excluded 86 OLLs patients. Eighty-five patients who had positive metal or dental materials on patch test reactions, localizing adjacent to, and in contact with lesions were diagnosed with OLCLs. One patient with chronic GVHD was diagnosed with OLL-GVHD. There were no OLDR patients due to systemic drugs. SCC did not develop in OLL patients. Seven hundred one (64.8%) patients were clinically and histopathologically diagnosed with OLP. Thereafter, 136 patients who were followed up for less than 6 months were excluded. Finally, 565 patients were analyzed in this study.

**Table 2:** Histopathological diagnosis of not OLP patients

Histopathological diagnosis		Cases (%)	Malignant transformation (%)
Ulcerative lesion		51 (17.3)	
Hyperkeratosis		47 (16.0)	
Epithelial dysplasia	mild	44 (14.9)	
	moderate	33 (10.9)	2/33 (0.6)
	severe	2 (0.7)	
Atypical epithelium		2 (0.7)	1/2 (50.0)
Stomatitis		41 (13.9)	
Subepithelial inflamation		37 (12.5)	
Granulation Tissue		6 (2.0)	
Gingivitis		6 (2.0)	
Pemphigoid		5 (1.7)	
Pemphigus		3 (1.0)	
Candidiasis		2 (0.7)	
Melanin pigmentation		1 (0.3)	
Fibulous polyp		1 (0.3)	
Squamous cell carcinoma		1 (0.3)	
Insufficient materials		13 (4.4)	
		294	3/294 (1.0)

### Criteria of the malignant transformation of OLP

The criteria of the malignant transformation of OLP were based on the criteria given by Idrees et al. [15]. The criteria were as follows: (1) the properly verified OLP diagnosis, (2) development of the cancerous lesion at the same site as the verified OLP lesion, and (3) follow-up duration of at least 6 months before SCC development.

### Sake index

To estimate the alcohol consumption, we used the sake index, calculated by multiplying the number of glasses (180 ml/glass) of sake per day by the number of years of drinking. In Japan, the sake index score ≥ 60 is considered to be a high-risk for developing oral cancer.

## Results

### Characteristics of OLP patients

Patient characteristics are summarized in Table 3. Of the 565 patients, 123 were male and 442 were female. The male: female ratio was 1:3.6. The patients were followed up for 6–220 months (mean ± standard deviation, 55.9 ± 45.3 months). The mean patient age at initial presentation was 60.5 ± 11.8 years (range, 21–93 years). One hundred eighteen (20.9%) patients had hypertension, 40 (7.1%) had diabetes mellitus, 31 (5.5%) had thyroid diseases, and 25 (4.4%) were seropositive for HCV. Eighteen (3.2%) patients had both hypertension and diabetes mellitus. Two patients (0.4%) had cutaneous LP, and no women had vulvovaginal lesions. Most patients had multiple oral sites of involvement. The most common site of involvement was buccal mucosa and gingiva (36.8%), followed by buccal mucosa (29.9%), gingiva (8.0%), buccal mucosa and tongue (6.2%), buccal mucosa, gingiva and tongue (4.8%), buccal mucosa and lips (3.7%), tongue (2.3%) (Table 4). Regarding the predominant clinical type, 325 (57.5%) patients had the red type (atrophic, erosive, bullous), and 240 (42.5%) had the white type (reticular, papular, plaque).

**Table 3:** Characteristics of OLP patients

		n	(%)
Gender	Male	123	(21.8)
	Female	442	(78.2)
Age	< 62	271	(48.0)
	≥ 62	294	(52.0)
Medical history	Hypertention	118	(20.9)
	Gastrointestinal disorder	65	(11.5)
	Diabetes mellitus	40	(7.1)
	Thyroid diseases	31	(5.5)
	Cardiovascular diseases	26	(4.6)
	Hepatitis C virus	25	(4.4)
	Depression	15	(2.7)
	Other liver diseases	8	(1.4)
	Cutaneous lichen planus	2	(0.4)
Clinical type	Red type	325	(57.5)
	White type	240	(42.5)
Candidal prevalence	Positive	194	(34.3)
	Negative	215	(38.1)
	Not examined	156	(27.6)

**Table 4:** Site distribution of OLP according to clinical type

Site	White type	Red type	Total (%)
Buccal mucosa, Gingiva	94	114	208 (36.8)
Buccal mucosa	82	87	169 (29.9)
Gingiva	22	23	45 (8.0)
Buccal mucosa, Tongue	11	24	35 (6.2)
Buccal mucosa, Gingiva, Tongue	8	19	27 (4.8)
Buccal mucosa, Lip	4	17	21 (3.7)
Tongue	7	6	13 (2.3)
Buccal mucosa, Gingiva, Lip	2	7	9 (1.6)
Buccal mucosa, Gingiva, Palate	2	4	6 (1.0)
Buccal mucosa, Palate	1	5	6 (1.0)
Buccal mucosa, Gingiva, Tongue, Palate	1	3	4 (0.7)
Buccal mucosa, Tongue, Lip	1	3	4 (0.7)
Buccal mucosa, Gingiva, Tongue, Lip	1	2	3 (0.5)
Gingiva, Tongue	1	2	3 (0.5)
Gingiva, Palate		2	2 (0.3)
Gingiva, Tongue, Lip		2	2 (0.3)
Buccal mucosa, Tongue, Lip, Palate		2	2 (0.3)
Buccal mucosa, Gingiva, Lip, Palate		1	1 (0.2)
Buccal mucosa, Gingiva, Tongue, Lip, Floor of the mouth		1	1 (0.2)
Buccal mucosa, Gingiva, Tongue, Floor of the mouth	1		1 (0.2)
Buccal mucosa, Gingiva, Floor of the mouth	1		1 (0.2)
Buccal mucosa, Floor of the mouth	1		1 (0.2)
Gingiva, Tongue, Palate		1	1 (0.2)
	240	325	565

The treatment of OLP was mostly performed using topical steroids, including 0.1% triamcinolone acetonide, to control inflammation and reduce painful symptoms. The topical steroids were applied once or twice daily depending on the severity of the lesions until complete remission or disappearance of symptoms and were resumed when the lesions or symptoms recurred. No side effects were observed during long-term treatment with topical steroids, except for oral candidiasis in 99 (17.5%) patients. *Candida* species were isolated from a swabbed sample from oral mucosa using conventional culture methods. The prevalence of oral candidiasis in OLP patients was 34.3%.

### Characteristics of the four patients with transformation of OLP to carcinoma

**Fig. 2:**
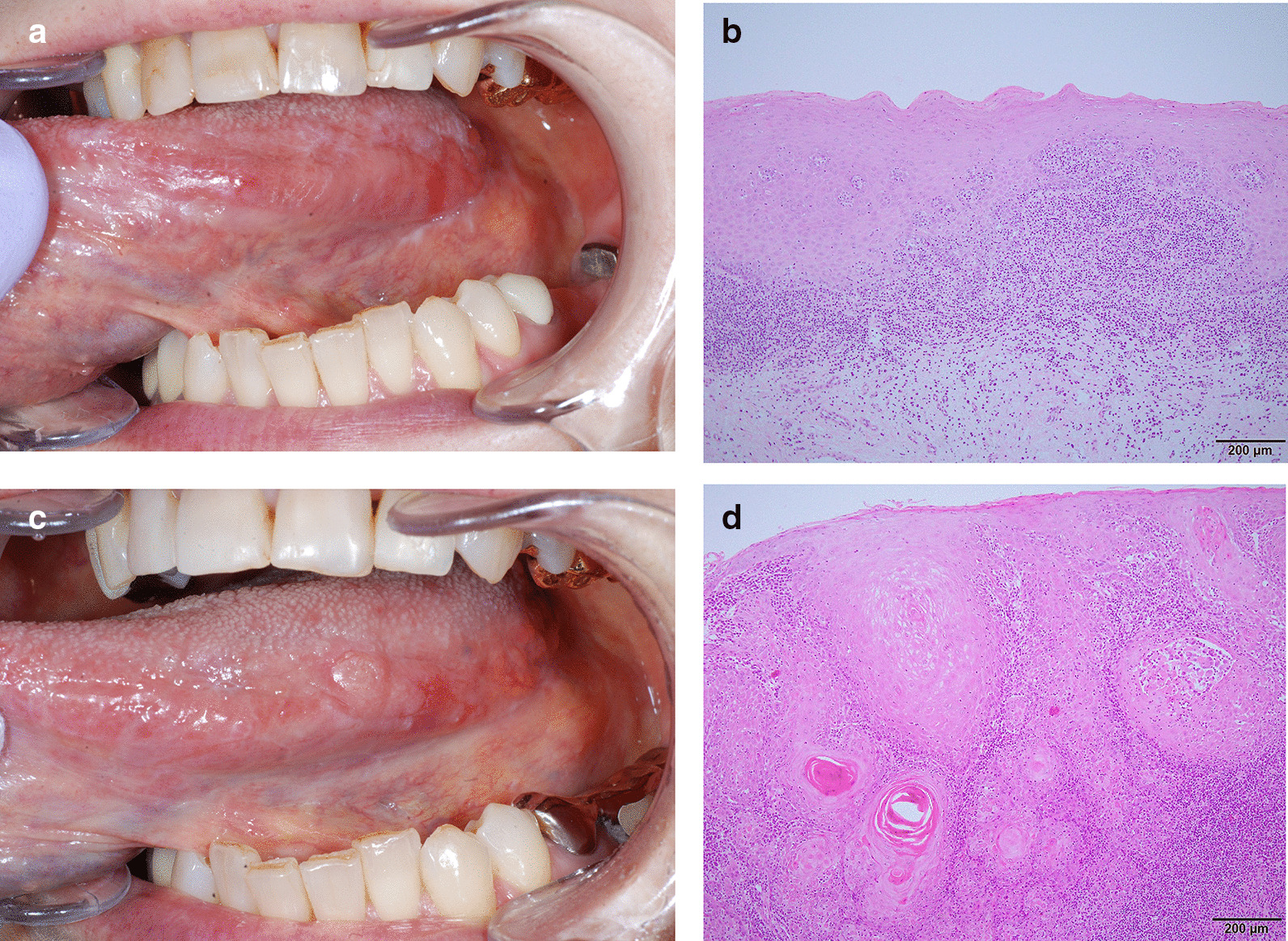
Malignant transformation in Case 4 showing clinical pictures and correlating histopathologic features in biopsy (haematoxylin–eosin staining) at initial presentation (**a**, **b**) and malignant transformation (**c**, **d**). **a** She had symmetric reticular and atrophic lesions on the bilateral buccal mucosa and tongue and showed no positive to metal patch test. The lesions did not improve with removal of the metal prosthesis close to the tongue and was clinically and histopathologically diagnosed with OLP. **b** Biopsy revealed histopathologic features of OLP, including parakeratosis with basal cell liquefactive degeneration, a band-like predominantly lymphocytic infiltrate adjacent to basal cells. **c** Two years and 5 months later, the lesion had transformed into SCC. **d** Biopsy revealed SCC

SCC developed in four patients (0.7%) at sites clinically and histopathologically diagnosed with OLP (Fig. 2). It was much lower than malignant transformation rate of 3.7% in oral epithelial dysplasia (OED) patients excluded from this study. There were no OLP patients who developed SCC from the site other than the biopsy site. One of the four patients with SCC was male, and three were female. The mean patient age at initial presentation was 65 ± 10.8 years. These 4 patients were HCV negative and without other serious medical problems. None of the patients had a history of smoking, but three patients had a history of alcohol drinking, one of whom was considered to be a high risk for developing oral cancer by sake index score of 74.1. The development occurred after a mean period of 52.5 ± 51.0 months (range, 25–129 months). The site of malignant transformation included the gingiva (n = 2), buccal mucosa (n = 1) and lateral tongue (n = 1). The clinical types of OLP in malignant lesions were three red type and one white type. The OLP with red type lesions developed SCC in shorter periods than white type lesions (Table 5).

**Table 5:** Characteristics of 4 patients with malignant transformation of OLP

No	Age	Medical history	Medication	Smoking	Alcohol drinking (type, average, daily dose, duration, sake index)	Transformation site	Clinical type	Candidal prevalence	Treatment (duration of topical steroids)	Follow-up period (months)
1	50 s	Ovarian cyst	None	Never	Never	Gingiva	White	Not examined	Topical steroids (11 months)	129
2	70 s	Sinusitis Spinal disc herniation Hemorrhoid	none	Never	Yes (Beer, 70 ml, 20y, 2.1)	Gingiva	Red	Not examined	Topical steroids (10 months)	25
3	70 s	Spinal canal stenosis	Calcium Vitamine D Minodronic Acid Hydrate Pregabalin	Never	Yes (Japanese distilled spirit, 180 ml, 40y, 74.1)	Buccal mucosa	Red	Negative	Topical steroids (3 months)	27
4	60 s	Depression	Zolpidem Clotiazepam	Never	Yes (Wine, 240 ml, 30y, 26.7)	Lateral tongue	Red	Positive	Topical steroids (5 months)	29

## Discussion

In this study, the malignant transformation rate of OLP was 0.7%. The malignant transformation rate cited in six recent systematic reviews and meta-analyses varied from 0.44 to 1.4% [9–14]. Our results were not much different from these results. However, Idrees et al. [14], showing the lowest malignant transformation rate of 0.44%, indicated that the rates cited by other authors were based on studies that included ineligible cases with nonconfirmed OLP, those with epithelial dysplasia at the initial diagnosis, and those with a follow-up duration of less than 6 months, thereby resulting a high malignant transformation rate of OLP. Their diagnostic criteria for OLP was based on the 2003 modified WHO criteria and the 2016 AAOMP proposed criteria. In fact, according to their diagnostic and malignant transformation criteria, the reports [15–17] of malignant transformation rate in patients with OLP similar number to this study they cited showed that malignant transformation rates were equal to or less than our rate [14]. Gonzales-Moles et al. [5] also suggested that the high incidence of malignant transformation described in many studies might be attributable to the misdiagnosis of some lesions as OLP. Therefore, the malignant transformation of OLP may be 1% or less.

The differentiation between OED and OLLs is important for the diagnosis of OLP. OED is a well-known precursor of SCC, and its presence and dysplasia grade influence the malignant transformation potential of OPMDs [18]. Iocca et al. [13] reported that the malignant transformation rate of OLP was the lowest in OMPDs, indicating the absence of epithelial dysplasia in OLP. In this study, 3 (3.7%) out of 81 OED patients developed SCC. This rate was considerably higher than the rate in patients with OLP, suggesting a low malignant potential of OLP. However, Lodi et al. [19] noted that lesions with clinical features of OLP but with dysplasia may represent an early phase in the malignant transformation of OLP. Thus, excluding OLP with epithelial dysplasia from these studies may still be debatable.

In this study, no OLL patients developed SCC. However, some studies have reported that OLCLs might possess malignant potential similar to that of OLP [20, 21]. Furthermore, Hougeir et al. suggested that contact allergy to dental metal restorations may be risk factor for development of SCC [22]. Therefore, regular follow-up is required for OLCL patients with malignant transformation as well as OLL-GVHD patients who are known to be at risk of SCC development. We did not find any OLDR suspected lesions because OLDR can occur at any time during the disease course, even more than 1 year after initiating medication. No standard diagnostic criteria for OLDR have been established, and further research on this subject is necessary.

Based on six recent systematic reviews and meta-analyses [11–14], tongue localization, red type (atrophic or erosive pattern), tobacco and alcohol consumption, and HCV infection significantly heighten the risk of the malignant transformation of OLP. In the present study, we could not investigate the clinical risk factors associated with malignant transformation due to the relatively small study population, which did not allow statistically meaningful analyses. However, we found that age 62 years and more, gingiva, and red-type OLP tended to have a higher risk of SCC development (Table 5). Further research on this subject is needed.

Regarding age and sex, the risk of the malignant transformation of OLP is believed to be higher in women than in men in the age group of 60–70 years [5]. Demographically, OLP is more common in women aged more than 40 years. Furthermore, Gonzales-Moles et al. [1] reported a significantly higher prevalence in subjects aged more than 50 years or more than 60 years. Thus, age and sex associated with the malignant transformation risk were suggested to be linked to demographics.

Regarding the clinical type and site, Aghbari et al. [10] reported that the rates of malignant transformation were 1.7%, 1.3%, and 0.1% in erosive, atrophic, and reticular patterns, respectively. The most common site was the tongue (1.05%), followed by the buccal mucosa (0.7%), the gingiva and the lips (0.6%), and the floor of the mouth (0.5%). With respect to the clinical type and site, the results of this study were almost consistent with previous results. In addition, the mean duration until malignant transformation was much shorter in those with red-type OLP than in those with white-type OLP. It has been suggested in the course of a chronic inflammatory process, cytokines can participate in malignant cell transformation, contributing to an increase in mutation rate. Interleukins such as IL-6, IL-17, or IL-23 contribute to tumor progression, and tumor necrosis factor (TNF)-α, transforming growth factor (TGF)-β, or IL-6 has a direct effect on the cell growth and survival rate [23]. Liu et al. [24] have suggested that inflammatory mediators such as cytokines and chemokines released from infiltrating T lymphocytes induce fundamental changes of proteins in oral epithelial cells, leading to the progression of OLP to SCC. Rhodus et al. [25] showed that elevated level of TNF-α, IL-1, IL-6, and IL-8 were found in the saliva of OLP patients. In addition, some studies showed that the expression of p53 and metalloproteinases (MMPs) in atrophic OLP were upregulated compared to nonatrophic OLP [26, 27]. Therefore, red-type OLP was suggested to have a higher malignant potential than white-type OLP.

Research has demonstrated a strong association between HCV infection and OLP, which is explained by the ability of the virus to replicate in oral mucosa cells and attract HCV-specific T lymphocytes [28]. Furthermore, HCV is an oncogenic virus and might be involved in oral carcinogenesis [29]. In this study, 4.4% of the OLP patients had an HCV infection; none developed SCC. Further studies on this subject are required.

The treatment of OLP involves the use of corticosteroids, cyclosporin, azathioprine, and retinoids. However, immunosuppressive agents may trigger malignant transformation, and the treatment of OLP patients with topical and/or systemic steroids did not influence the risk of malignant transformation [19]. In this study, the mean duration of topical steroids treatment before SCC development was 7.3 ± 3.9 months (range; 3–11 months). There were also no cases of continuous use of topical steroids for a long period of time. Thus, in this study, the treatment did not affect the risk of malignant transformation.

OLP patients have an increased prevalence of Candida infection and are predisposed to candidiasis with topical or systemic immunosuppressive therapy, however no study assesses the presence of Candida in OLP cases with SCC [11]. Candida generates chronic inflammation and can produce carcinogenic N-nitrosobenzylmethylamine [30] and mutagenic amounts of acetaldehyde [31]. In this study, 1 out of 2 red-type OLP with Candida infection developed SCC. Further studies on this subject are needed.

## Conclusions

Our results showed a malignant transformation rate of 0.7%, suggesting that OLP is associated with a low risk of malignant transformation. Therefore, we recommend regular follow-up for patients with OLP and clear differentiation of OED and OLLs to enable early detection of malignant transformation. Further investigation of the clinical risk factors associated with malignant transformation of OLP based on accurate exclusion and inclusion criteria is needed.

## Data Availability

The data used during this article are available from the corresponding author on reasonable request.

## Abbreviations

AAOMP: American Academy of Oral and Maxillofacial Pathology
HCV: Hepatitis C virus
OLCLs: Oral lichenoid contact lesions
OLDRs: Oral lichenoid drug reactions
OLL-GVHD: Oral lichenoid lesions of graft-versus-host disease
OLLs: Oral lichenoid lesions
OLP: Oral lichen planus
OPMD: Oral potentially malignant disorder
SCC: Squamous cell carcinoma
WHO: World Health Organization
